# The Genomic Profile Associated with Risk of Severe Forms of COVID-19 in Amazonian Native American Populations

**DOI:** 10.3390/jpm12040554

**Published:** 2022-04-01

**Authors:** Lucas Favacho Pastana, Thays Amâncio Silva, Laura Patrícia Albarello Gellen, Giovana Miranda Vieira, Letícia Almeida de Assunção, Luciana Pereira Colares Leitão, Natasha Monte da Silva, Rita de Cássia Calderaro Coelho, Angélica Leite de Alcântara, Lui Wallacy Morikawa Souza Vinagre, Juliana Carla Gomes Rodrigues, Diana Feio da Veiga Borges Leal, Marianne Rodrigues Fernandes, Sandro José de Souza, José Eduardo Kroll, André Mauricio Ribeiro-dos-Santos, Rommel Mario Rodríguez Burbano, João Farias Guerreiro, Paulo Pimentel de Assumpção, Ândrea Campos Ribeiro-dos-Santos, Sidney Emanuel Batista dos Santos, Ney Pereira Carneiro dos Santos

**Affiliations:** 1 Laboratório do Núcleo de Pesquisa em Oncologia, Universidade Federal do Pará, Belém 66073-000, Brazil; lucas.pastana@ics.ufpa.br (L.F.P.); thaysamnc@gmail.com (T.A.S.); laura.patricia.agellen@hotmail.com (L.P.A.G.); giovanamirandav@gmail.com (G.M.V.); leticiaalmeidaenf96@gmail.com (L.A.d.A.); colares.luciana@gmail.com (L.P.C.L.); ntshmonte@gmail.com (N.M.d.S.); rccalderarocoelho@gmail.com (R.d.C.C.C.); angelica.alcantara99@gmail.com (A.L.d.A.); luivinagre@gmail.com (L.W.M.S.V.); julianacrodrigues@gmail.com (J.C.G.R.); dianafeio@hotmail.com (D.F.d.V.B.L.); fernandesmr@yahoo.com.br (M.R.F.); rommelburbano@gmail.com (R.M.R.B.); assumpcaopp@gmail.com (P.P.d.A.); sidneysantosufpa@gmail.com (S.E.B.d.S.); 2 Instituto do Cérebro, Universidade Federal do Rio Grande do Norte, Natal 59076-550, Brazil; sandro@neuro.ufrn.br (S.J.d.S.); jkpenga@gmail.com (J.E.K.); 3 BioME, Universidade Federal do Rio Grande do Norte, Natal 59078-400, Brazil; 4 Institute of Systems Genetics, West China Hospital, University of Sichuan, Chengdu 610041, China; 5 Laboratório de Genética Humana e Médica, Universidade Federal do Pará, Belém 66075-110, Brazil; andremrsantos@gmail.com (A.M.R.-d.-S.); joao.guerreiro53@gmail.com (J.F.G.); akelyufpa@gmail.com (Â.C.R.-d.-S.)

**Keywords:** COVID-19, gene, risk factor, genetic variant

## Abstract

Genetic factors associated with COVID-19 disease outcomes are poorly understood. This study aimed to associate genetic variants in the *SLC6A20*, *LZTFL1*, *CCR9*, *FYCO1*, *CXCR6*, *XCR1*, and *ABO* genes with the risk of severe forms of COVID-19 in Amazonian Native Americans, and to compare the frequencies with continental populations. The study population was composed of 64 Amerindians from the Amazon region of northern Brazil. The difference in frequencies between the populations was analyzed using Fisher’s exact test, and the results were significant when *p* ≤ 0.05. We investigated 64 polymorphisms in 7 genes; we studied 47 genetic variants that were new or had impact predictions of high, moderate, or modifier. We identified 15 polymorphisms with moderate impact prediction in 4 genes (*ABO*, *CXCR6*, *FYCO1*, and *SLC6A20*). Among the variants analyzed, 18 showed significant differences in allele frequency in the NAM population when compared to others. We reported two new genetic variants with modifier impact in the Amazonian population that could be studied to validate the possible associations with COVID-19 outcomes. The genomic profile of Amazonian Native Americans may be associated with protection from severe forms of COVID-19. This work provides genomic data that may help forthcoming studies to improve COVID-19 outcomes.

## 1. Introduction

The coronavirus disease 2019 (COVID-19) outbreak started when a few patients were hospitalized with acute respiratory distress syndrome in December 2019. At the end of January 2020, a total of 1975 COVID-19 cases were confirmed in China, with a total of 56 deaths [1]. The new infection spread worldwide, and the World Health Organization (WHO) declared COVID-19 a pandemic on 11 March 2020 [2]. Globally, the number of confirmed cases of COVID-19 has reached almost 386,548,962, including 5,705,754 deaths as of 6 February 2022 [3]

All individuals are susceptible to COVID-19 infection; however, the severity of the disease varies significantly between individuals and populations. There are many host, viral, and environmental factors contributing to the COVID-19 phenotype [4]; however, the genetic factors associated with COVID-19 disease outcomes are poorly understood. The discovery of human genetic factors associated with this disease’s severity would be invaluable in identifying high-risk groups, and would enable the stratification of individuals according to risk in order to guide personalized prevention and therapeutics [5].

Infectious diseases continue to disproportionately affect indigenous peoples and admixture populations with Amerindian ancestry [6,7,8,9,10], and COVID-19 has reached indigenous communities [11,12,13,14,15,16]. This population has a particular genetic vulnerability to infection due to different frequencies of alleles in immune system genes [17]. Their high genetic homozygosity has been suggested to be a consequence of a serial founder effect, compounded by successive generations of inbreeding [18]. This genetic factor may result in a significant loss of diversity and have consequences on health and performance [8].

In genome-wide association studies (GWASs), the identification of potential genetic factors associated with the development of COVID-19 has been investigated. The first GWAS analysis with 1980 patients with COVID-19 identified two loci associated with the most severe forms of COVID-19: one locus was 3p21.31, which includes the genes *SLC6A20*, *LZTFL1*, *CCR9*, *FYCO1*, *CXCR6*, and *XCR1*, while the other was 9q34.21, including the *ABO* blood group. These results might explain the heterogeneity of the disease [19].

The *CXCR6*, *CCR9*, and *XCR1* genes are chemokine receptors, and directly participate in the functioning of cells of the immune system and the expression of interleukins. Other selected genes have more heterogeneous actions. The *LZTFL1* gene plays a role in intracellular signaling actions. The *FYCO1* gene is involved in the transport of autophagic vesicles, the *SLC6A20* gene activates virus adhesion co-receptors in the cell, and the *ABO* gene is related to glycosylation of the H antigen for the formation of blood group variability [19].

Subsequently, Shelton et al. identified a strong association between blood type and COVID-19 diagnosis. Moreover, variants on chromosome 3p21.31 were strongly associated with COVID-19 outcome severity [20]. The present study investigated genetic variants in the *SLC6A20*, *LZTFL1*, *CCR9*, *FYCO1*, *CXCR6*, *XCR1*, and *ABO* genes that are potentially related to severe forms of COVID-19 in Amazonian Native American populations, and compared their frequencies with continental populations.

## 2. Materials and Methods

### 2.1. Study and Reference Populations

All participants in the study and their ethnic group leaders signed written informed consent. The recruitment period was before the COVID-19 pandemic, from September 2017 to December 2018. The study was approved by the National Committee for Ethics in Research (CONEP) and the Research Ethics Committee of the UFPA Tropical Medicine Center under CAAE number 20654313.6.0000.5172, and by the Research Ethics Committee of the UFPA under project 123/98.

The Amazonian Native American (NAM) cohort was composed of 64 Amerindians from the Amazon region of northern Brazil. The NAM population was healthy and did not share family relationships. The genetic ancestry was obtained through a panel of 61 ancestry-informative markers (AIMs), which were used for estimating individual ancestry and admixture from three continents (European, African, and Amerindian) in three multiplex PCR reactions [7,21,22]. The amplicons were analyzed by electrophoresis using the ABI Prism 3130 sequencer and the GeneMapper ID v.3.2 software. The individual proportions were estimated using STRUCTURE v.2.3.3.

The allele frequencies of the NAM population were obtained directly by gene counting and compared with continental populations (available at http://www.1000genomes.org). The 1000 Genomes project dataset corresponds to full-length DNA sequences from 2504 human individuals that include 26 population groups clustered into 5 larger population groups (African: AFR; American: AMR; East Asian: EAS; European: EUR; and South Asian: SAS). These populations include 661 individuals of African (AFR), 347 of American (AMR), 504 of East Asian (EAS), 503 of European (EUR), and 489 of South Asian (SAS) descent.

For the samples with European, East Asian, and South Asian ancestry, populations across the geographic range had ~1% FST; populations from Africa were related to the Yoruba and, therefore, not a comprehensive representation of Africa; for populations in the Americas, the samples were from two populations with primarily African and European ancestry—people with African Ancestry in the southwest USA (ASW), and those of Afro-Caribbean descent in Barbados (ACB)—and four populations (people with Mexican Ancestry in Los Angeles, CA, USA (MXL), Colombians in Medellin, Colombia (CLM), Puerto Ricans in Puerto Rico (PUR), and Peruvians in Lima, Peru (PEL)) with a wide range of European, African, and Indigenous American ancestry were chosen to represent the wide variation in ancestry proportions observed in North, Central, and South America.

### 2.2. Extraction of DNA and Preparation of the Exome Library

The DNA extraction was performed via the phenol–chloroform method [23]. The quantification and integrity of genetic material were analyzed using a NanoDrop 8000 spectrophotometer (Thermo Fisher Scientific Inc., Wilmington, DE, USA) and electrophoresis in 2% agarose gel, respectively.

The exome libraries were prepared using the Nextera Rapid Capture Exome (Illumina^®^, San Diego, CA, USA) and SureSelect Human All Exon V6 (Agilent, Santa Clara, CA, USA) kits. The sequencing reactions were run on the NextSeq 500^®^ platform (Illumina^®^, San Diego, CA, USA) using the NextSeq 500 High-Output v2 300 cycle kit (Illumina^®^, San Diego, CA, USA).

### 2.3. Bioinformatic Analysis

The quality of the FASTQ reads was analyzed (FastQC v.0.11—http://www.bioinformatics.babraham.ac.uk/projects/fastqc/; accessed on 20 January 2022), and the samples were filtered to eliminate low-quality readings (fastx_tools v.0.13—http://hannonlab.cshl.edu/fastx_toolkit/; accessed on 20 January 2022). The sequences were mapped and aligned with the reference genome (GRCh38) using the BWA v.0.7 tool (http://bio-bwa.sourceforge.net/; accessed on 20 January 2022). Following this alignment with the reference genome, the file was indexed and sorted (SAMtools v.1.2—http://sourceforge.net/projects/samtools/; accessed on 20 January 2022). Subsequently, the alignment was processed for duplicate PCR removal (Picard Tools v.1.129—http://broadinstitute.github.io/picard/; accessed on 20 January 2022), mapping quality recalibration, and local realignment (GATK v.3.2—https://www.broadinstitute.org/gatk/; accessed on 20 January 2022). The results were processed in order to determine the variants from the reference genome (GATK v.3.2). The analysis of the variant annotations was carried out using the ViVa1 (Viewer of Variants) software developed by the Federal University of Rio Grande do Norte (UFRN)’s bioinformatics team. The databases and their versions used for variant annotations were SnpEff v.4.3.T, Ensembl Variant Effect Predictor (Ensembl release 99), and ClinVar (v.2018-10). For in silico prediction of pathogenicity, we used SIFT (v.6.2.1), PolyPhen-2 (v.2.2), LRT (November 2009), Mutation Assessor (v.3.0), Mutation Taster (v. 2.0), FATHMM (v.2.3), PROVEAN (v.1.1.3), MetaSVM (v1.0), M-CAP (v1.4), and FATHMM-MKL (http://fathmm.biocompute.org.uk/about.html; accessed on 20 January 2022). More information about bioinformatic analyses is described in the works of Rodrigues et al. (2020) and Ribeiro-dos-Santos et al. (2020) [22,24].

### 2.4. Statistical Analyses

The difference in frequencies between the populations was analyzed using Fisher’s exact test, and the results were significant when *p* ≤ 0.05. The interpopulation variability of the polymorphisms was assessed using Wright’s fixation index (FST). These analyses were performed using RStudio v.4.1.0.

### 2.5. Selection of Variants

Seven genes (*SLC6A20*, *LZTFL1*, *CCR9*, *FYCO1*, *CXCR6*, *XCR1*, and *ABO*) used in recent GWAS studies were selected. These studies identified genes at the loci 3p21.31 and 9q34.21 as being likely related to disease severity [19,20,25]. For subsequent analyses, the selection of variants was based on three main criteria: (a) a minimum of 10 reads of coverage (fastx_tools v.0.13-http://hannonlab.cshl.edu/fastx_toolkit/; accessed on 20 January 2022), (b) the variant should have an allele frequency described in all continental populations from the 1000 Genomes Project Consortium [10], and (c) the variant impact should be either modifier, moderate, or high, according to SnpEff classification (https://pcingola.github.io/SnpEff/; accessed on 20 January 2022)—a program that predicts coding effects such as synonymous or non-synonymous amino acid replacement, start codon gains or losses, stop codon gains or losses, and/or frameshifts. Predicted effects concerned protein-coding genes [26]. A total of 64 variants were found in the *ABO*, *CCR9*, *CXCR6*, *FYCO1*, *LZTFL1*, *XCR1*, and *SLC6A20* genes, and are described in Supplementary Table S1. The analyses were directed to 38 variants that met all specifications of the selection criteria. The function of these genes is summarized in Table 1.

## 3. Results

In our study, we identified 64 polymorphisms distributed in 7 genes: 14 of them from the *ABO* gene, 3 from the *CCR9* gene, 1 from the *CXCR6* gene, 1 from the *XCR1* gene, 11 from the *SLC6A20* gene, 4 from the *LZTFL1* gene, and 30 from the *FYCO1* gene (Supplementary Table S1). Among these 64 variants, only 47 were new variants or had impact prediction by SnpEff of high, moderate, or modifier. A total of 10 SNPs were located in the *ABO* gene, 2 in the *CCR9* gene, 1 in the *CXCR6* gene, 21 in the *FYCO1* gene, 4 in the *LZTFL1* gene, and 8 in the *SLC6A20* gene.

These variants are described in Table 2, which contains characteristics including SNP ID, chromosomal region, nucleotide change, SnpEff software impact prediction, and allele frequency for the NAM population and the five continental populations present in the 1000 Genomes platform (AFR, AMR, EAS, EUR, and SAS).

We identified three new variants in the Amazonian Native American population: One of the variants was located in the *ABO* gene at position 133262062, with base exchange C > A, in the intronic region, with an allele frequency of 0.016. The second variant was identified in the *FYCO1* gene at position 45959401, with base exchange G > A, in the exonic region, with low impact predicted by SnpEff and an allele frequency of 0.018. The third variant was identified in the *LZTFL1* gene at position 45827235, with a TCTG > T deletion, in the intronic region, and with an allele frequency of 0.016.

Among the polymorphisms analyzed, eight showed no variant allele frequency in Amerindian populations: two of these were in the *ABO* gene (rs200932155 and rs8176721), one in the *CCR9* gene (rs17764980), two in the *FYCO1* gene (rs3796376 and rs13069079), one in the *LZTFL1* gene (rs1129183), and two in the *SLC6A20* gene (rs61731475 and rs139429025).

Regarding the classification based on impact forecast by SnpEff, the polymorphism in the *ABO* gene (rs55727303) presented a high impact with a significant difference in all of the correlations. We identified 15 polymorphisms with moderate impact prediction in 4 genes, distributed in 4 polymorphisms in the *ABO* gene (rs8176748, rs8176740, rs8176720, and rs1053878), 1 in the *CXCR6* gene (rs2234355), 8 in the *FYCO1* gene (rs3796375, rs35678722, rs113517878, rs4683158, rs13079478, rs13059238, rs33910087, and rs3733097), and 2 in the *SLC6A20* gene (rs140440513 and rs17279437).

Additionally, 38 variants had allele frequency in all populations—a necessary requirement for comparative analysis between the populations studied using Fisher’s exact test (Table 2). The remaining variants were excluded because they had no frequency description in the 1000 Genomes Project.

Among the variants analyzed, 18 showed significant differences of the NAM population when compared to all other continental populations (AFR, AMR, EAS, EUR, and SAS): 7 belonging to the *ABO* gene (rs55727303, rs8176748 rs8176740, rs8176720, rs2073824, rs559723, and rs616154), 7 from the *FYCO1* gene (rs3733100, rs3796375, rs35678722, rs113517878, rs1532071, rs3733097, and rs751552), 2 from the *SLC6A20* gene (rs2252547 and rs140440513), 1 from the *LZTFL1* gene (rs141398338), and 1 from the *CCR9* gene (rs147314165). The remaining 20 variants that were not significant in any comparisons are described in Table 3.

**Figure 1:**
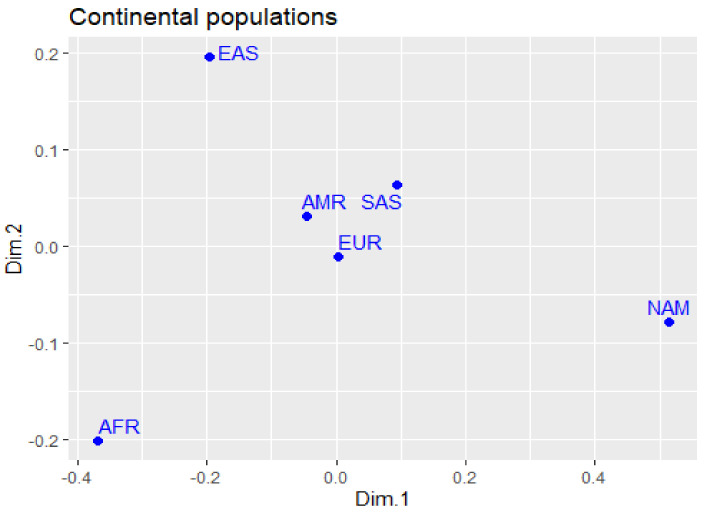
Differences in allele frequencies of the variants studied in the continental populations and the Native American population, plotted in MDS.

Figure 1 represents the genomic differences between the studied populations in multidimensional scaling (MDS), based on the fixation index (FST) of polymorphisms. We can observe the presence of a genetically similar core set composed of the AMR, EUR, and SAS components, while the other groups (NAM, EAS, and AFR) are at extreme points of the graph.

## 4. Discussion

COVID-19 presents a new threat to the health of Native Amerindians living remotely. In the Amazon region, it is estimated that there are around 78 Amerindian tribes living in isolation [44]. The Amerindian people belong to a vulnerable population, who lack immunity to many infectious diseases [8]. In Brazilian territory, there were 59,574 cases and 871 deaths recorded in Amerindians as of 6 February 2022 [16].

The occurrence of numerous stochastic events—such as geographic isolation, inbreeding, and genetic drift—may contribute to genetic differentiation in Amazonian Native American populations [45,46,47,48,49]. These variables influence the formation of indigenous peoples, their ethnic structure and, consequently, their genomic patterns, with different allele frequencies from other continental populations [50,51].

The genomic differences and distinct sociodemographic and anthropological characteristics of Amerindian populations are related to epidemiological differences in respiratory and viral diseases—such as tuberculosis (TB), human immunodeficiency virus (HIV), human T-lymphotropic virus (HTLV), and human herpesvirus type 8 (HHV-8)—observed in comparisons between Amerindian and non-Amerindian populations. Studies suggest that genomic alterations in the immune response patterns and the parasite–host molecular interaction may be the causes of the observed differences [7,17,52].

GWA studies are widely used in the identification of genetic variants associated with complex and multifactorial diseases, such as cardiovascular, psychiatric, infectious, and numerous other diseases [53,54,55]. The first GWA study investigated the multigenic group of chromosome 3 in patients infected with COVID-19 [19]. The 3p21.31 locus has the *XCR1*, *CCR9*, *CXCR6*, *SLC6A20*, *LZTFL1*, and *FYCO1* genes, in which rs11385942 (*LZTFL1*) and rs657152 (*ABO*) were significantly associated with severe forms of the disease [19].

In Shelton’s 2021 study, the variants related to disease severity and susceptibility were rs13078854 of the *LZTFL1* gene and rs9411378 of the *ABO* gene [20]. These findings reinforce the importance of the 3p21.31 locus and the 9q34.21 locus—particularly with regard to the *ABO* and *LZTFL1* genes; therefore, we focus our discussion on these two genes.

The association between the *ABO* blood group and COVID-19 infection and severity was studied. Blood type A might be more susceptible to COVID-19 infection, while blood type O might be less susceptible to this disease. [19]. In other epidemiological and genomic studies, susceptibility was also lower in O blood group patients [20,56,57]. The O allele is the most frequent blood type found in the Native American population [51,58]. In addition, the *ABO* blood group has previously been linked to susceptibility to other diseases, such as influenza, malaria, schistosomiasis, and SARS-CoV-2 [59].

Some proposed mechanisms for the association between ABO blood type and SARS-CoV-2 infection were investigated, as follows: (1) anti-A and/or anti-B antibodies play a role as viral neutralizing antibodies when binding to A and/or B antigens expressed on the viral envelope; (2) the SARS-CoV-2 S protein is bound by human anti-A antibodies, and prevents entry into the lung epithelium when blocking the interaction between the virus and ACE2R; (3) an increase in ACE-1 activity in group A individuals can cause predisposition to cardiovascular disease and lead to severe COVID-19; (4) ABH glycans in the SARS-CoV-2 S protein may modify cellular receptors of SARS-CoV-2 for ACE2R; (5) ABH glycans on target cells could serve as alternative, lower affinity receptors for the SARS-CoV-2 S protein, or could bind other viral envelope structures [42,60,61].

In our study, significant differences were found in the majority of polymorphisms in the *ABO* gene between the Amerindian populations and continental populations. We hypothesize that the differences in genomic profiles and the novel variants identified in the Native American population may influence the development of severe forms of COVID-19. However, further studies will be needed in COVID-19-positive individuals in this population in order to better understand the potential influence of these variants on this infection.

The *LZTFL1* gene encodes the leucine zipper transcription factor-like 1, and its function is related to tumor-suppressor action and negative regulation of the hedgehog signaling pathways. In knockout zebrafish (*Danio rerio*) experimental models, impaired cell traffic in ciliary membranes, retinal degeneration, and obesity were observed [28]. In addition, this gene has high expression in lung tissues; however, the mechanisms directly related to SARS-CoV-2 infection remain unknown [25].

In our study, we found four variants related to the *LZTFL1* gene, and only rs141398338 showed a significant difference in the NAM population when compared to the continental populations. There have been no reports in the literature on the association between rs141398338 and severe forms of COVID-19. New variants identified in the Native American population may influence the development of severe forms of COVID-19, and further human genetic studies need to be carried out in order to clarify this issue.

In addition, we identified three new variants in the Amazonian NAM population; these SNPs were located in the *FYCO1*, *ABO*, and *LZTFL1* genes. The first has low clinical impact, while the following two have modifier impact. These mutations—especially the ones with modifier impact—could have important potential as markers of severe forms of COVID-19 in Amazonian indigenous populations, as well as intronic regional mutations that significantly influence gene expression levels [62]. Larger studies should be performed to confirm these new variants in patients diagnosed with COVID-19.

Genetic variants associated with severe COVID-19 indicated by the studies of Ellinghaus and Shelton et al. [19,20] were not found in the Amazonian Native American population. The allelic frequencies of the SNPs in the NAM group were lower than for any of the other groups in our study, showing that the Amazonian Native Americans have low genetic variability and a different genetic pool. Genetic variants present in the NAM population and low genetic variability could indicate a protective factor against severe COVID-19.

The limitation of our study was the small number of NAM individuals, who come from isolated and relatively small populations in the Amazonian region. This study is a preliminary severe COVID-19 study, and did not investigate individuals with COVID-19 infection. We collected blood samples from individuals before the COVID-19 pandemic. Our results may reveal important information and contribute to the assessment of individual risk for the development of this disease.

## 5. Conclusions

Genetic variants associated with severe COVID-19 were not found in the Amazonian Native American population. The allele frequency for the candidate genes in the NAM group was significantly different from the frequencies observed in continental groups. This may provide a protective factor against severe COVID-19. We also identified two new genetic variants with modifier impact in the Amazonian population that could be studied in order to validate the possible associations with COVID-19 outcomes. This work contributes to the elucidation of the genomic profile of Amazonian Native Americans—an understudied population—by providing genomic data that may help forthcoming studies to improve COVID-19 outcomes. Future studies should be performed in this population to identify more genetic variants related to severe COVID-19.

## Figures and Tables

**Table 1:** Function of the *SLC6A20,* *LZTFL1*, *CCR9, CXCR6,* *XCR1, FYCO1,* and *ABO* genes.

Gene	Description *
*SLC6A20*	This gene encodes the protein sodium–amino acid (proline) transporter 1 (SIT1), which interacts with the angiotensin-converting enzyme 2 (*ACE2*)—the SARS-CoV-2 cell-surface receptor—allowing its heterodimerization [19]. The heterodimerization of the *ACE2* protein is necessary for the formation of a quaternary structure that functions as a binding site for the SARS-CoV-2 protein S [27].
*LZTFL1*	The *LZTFL1* gene encodes the leucine zipper transcription factor-like 1, and its function is related to tumor-suppressor action and negative regulation of the hedgehog signaling pathways. This gene has high expression in lung tissues [25,28]; it is related to the functioning of the cilia of the pulmonary epithelium and to the signaling of important intracellular pathways, regulating the epithelial–mesenchymal transformation [29].
*CCR9*	CC chemokines are mainly responsible for the recruitment of lymphocytes. *CCR9* is the receptor for the C-C chemokine ligand 25 (CCL25). The CCR9 receptor is mainly found on immature T lymphocytes and the surface of intestinal cells [30]. Animal studies have shown that the CCR9/CCL25 complex participates in the action of T helper 1 (Th1) cells. Another finding indicates that in knockout rats there was a reduction in the mRNA levels of pro-inflammatory cytokines (i.e., IL-6, IL-1β, and TNF-α) [30,31].
*CXCR6*	CXC chemokines have the highest ability to attract neutrophils and monocytes (30). *CXCR6* is the receptor for *CXCL16*; in cellular studies and animal models, it has been shown to regulate inflammatory activity and influence the levels of INF-γ and TNF-α secreted by CD4+ T cells [32,33].
*XCR1*	*XCR1* encodes the receptor of the XCL-1 ligand. The receptor triggers chemotactic signals in the presence of the ligand [34]. *XCR1* is expressed in the lung tissue. Further reports suggest that *XCL1* expression in NK cells and CD8+ T cells is constitutively detectable at a steady state, and is elevated during viral infection in mice and humans. The XCL1–XCR1 axis is important for efficient cytotoxic immune response mediated by CD8+ T cells [35].
*FYCO1*	This gene is responsible for the production of a Rab7 adapter protein, and has the function of assisting in the intracellular transport of autophagic vesicles via transport by microtubules. To carry out the transport, the encoded protein interacts with Rab7 GTPase, phosphatidylinositol-3-phosphate (PI3P), the autophagosome marker LC3, and the kinesin KIF5 [36,37]; it was previously found to be related to inclusion body myositis (IBM) and autosomal recessive congenital cataracts (CATC2) [38,39].
*ABO*	The *ABO* gene encodes the enzyme alpha 1-3-galactosyltransferase, which transforms the H antigen expressed on the cell surface of several cell types into A and B antigens. Furthermore, the enzyme converts the H antigen into the von Willebrand factor [40]. Studies indicate that group A confers risk of developing severe forms of infection, while group O confers protection [1]. This effect is related to the expression of anti-A and anti-B antibodies that could neutralize the interaction of the virus protein S with ACE2, blocking its adsorption [41]. Another hypothesis would be its action in the formation of the von Willebrand factor and its relationship with its expression in the pulmonary endothelium, indirectly influencing pro-inflammatory regulation and cell adhesion [42,43].

* The gene functions related to COVID-19 are hypotheses raised by other authors.

**Table 2:** Description of the variants with predicted high, moderate, or modifier impact, and new variants for the genes *ABO*, *CCR9*, *CXCR6*, *FYCO1*, *LZTFL1*, and *SLC6A20*.

Gene	Position	SNP ID	Ref ^a^	Var ^b^	Impact Predicted by SnpEff	Variant Allele Frequency					
						NAM	AFR	AMR	EAS	EUR	SAS
*ABO*	133256189	rs55727303	C	T	High	0.281	0.001	0.108	-	0.012	0.026
*ABO*	133255902	rs8176748	C	T	Moderate	0.814	0.243	0.431	0.290	0.221	0.220
*ABO*	133256085	rs8176740	A	T	Moderate	0.814	0.242	0.431	0.289	0.221	0.220
*ABO*	133256264	rs1053878	G	A	Moderate	0.016	0.256	0.068	0.151	0.099	0.029
*ABO*	133257465	rs8176721	G	A	Moderate	0.000	0.155	0.017	-	0.005	-
*ABO*	133257486	rs8176720	T	C	Moderate	0.814	0.489	0.494	0.483	0.337	0.464
*ABO*	133257246	rs2073824	A	G	Modifier	0.728	0.469	0.496	0.549	0.336	0.465
*ABO*	133257320	rs2073825	A	T	Modifier	0.235	0.241	0.431	0.289	0.222	0.225
*ABO*	133262062	*	C	A	Modifier	0.016	-	-	-	-	-
*ABO*	133275050	rs616154	C	T	Modifier	0.031	0.531	0.408	0.408	0.535	0.631
*ABO*	133275068	rs559723	A	G	Modifier	0.184	0.531	0.408	0.408	0.536	0.631
*CCR9*	45894830	rs7648467	C	A	Modifier	0.014	0.448	0.050	-	0.013	0.008
*CCR9*	45897524	rs17764980	G	A	Modifier	0.000	0.005	0.058	0.004	0.120	0.383
*CXCR6*	45946488	rs2234355	G	A	Moderate	0.033	0.491	0.068	-	0.005	0.001
*FYCO1*	45959401	*	G	A	Low	0.019	-	-	-	-	-
*FYCO1*	45923752	rs35678722	G	A	Moderate	0.083	0.012	0.001	-	-	-
*FYCO1*	45966331	rs13079478	G	T	Moderate	0.070	0.005	0.059	0.004	0.122	0.360
*FYCO1*	45966333	rs13059238	T	C	Moderate	0.070	0.019	0.063	0.004	0.123	0.359
*FYCO1*	45966722	rs113517878	C	T	Moderate	0.083	0.003	0.003	-	-	-
*FYCO1*	45967298	rs3796375	G	A	Moderate	0.822	0.093	0.565	0.661	0.431	0.372
*FYCO1*	45967995	rs33910087	G	A	Moderate	0.070	0.017	0.059	0.005	0.122	0.359
*FYCO1*	45968372	rs3733100	C	G	Moderate	0.885	0.210	0.643	0.667	0.556	0.731
*FYCO1*	45968585	rs4683158	C	T	Moderate	0.994	0.982	0.914	1.000	0.801	0.922
*FYCO1*	45979767	rs1306733846	C	T	Moderate	0.000	0.000	0.000	0.000	0.000	0.000
*FYCO1*	45923467	rs6800954	C	T	Modifier	0.143	0.287	0.193	0.307	0.216	0.148
*FYCO1*	45936407	rs1873002	T	C	Modifier	1.000	1.000	1.000	1.000	1.000	1.000
*FYCO1*	45938385	rs9875616	G	A	Modifier	0.859	0.914	0.850	0.954	0.746	0.879
*FYCO1*	45959378	rs13069079	G	A	Modifier	0.000	0.005	0.058	0.004	0.121	0.371
*FYCO1*	45959571	rs1532071	G	A	Modifier	0.908	0.260	0.614	0.652	0.529	0.732
*FYCO1*	45959735	rs76597151	G	A	Modifier	0.014	0.017	0.062	0.004	0.122	0.371
*FYCO1*	45969944	rs17214952	A	G	Modifier	0.014	0.019	0.063	0.004	0.123	0.360
*FYCO1*	45973053	rs41289622	T	G	Modifier	0.070	0.005	0.059	0.004	0.122	0.361
*FYCO1*	45975359	rs751552	A	T	Modifier	0.814	0.063	0.565	0.661	0.431	0.371
*FYCO1*	45981341	rs36122610	G	A	Modifier	0.054	0.005	0.059	0.004	0.122	0.358
*FYCO1*	45984767	rs3733097	G	A	Modifier	0.853	0.067	0.561	0.655	0.432	0.372
*LZTFL1*	45828480	rs1129183	C	T	Moderate	0.000	0.043	0.038	0.039	0.074	0.077
*LZTFL1*	45827235	*	TCTG	T	Modifier	0.016	-	-	-	-	-
*LZTFL1*	45842023	rs138230559	C	T	Modifier	0.009	0.033	-	-	-	-
*LZTFL1*	45842083	rs141398338	A	C	Modifier	0.083	0.005	-	-	0.002	-
*SLC6A20*	45759079	rs140440513	C	T	Moderate	0.083	0.000	0.000	0.000	0.000	0.000
*SLC6A20*	45759901	rs61731475	T	C	Moderate	0.000	-	0.006	-	0.014	-
*SLC6A20*	45772602	rs17279437	G	A	Moderate	0.017	0.005	0.043	0.006	0.092	0.031
*SLC6A20*	45775922	rs139429025	T	C	Moderate	0.000	0.012	-	-	-	-
*SLC6A20*	45758379	rs2251347	C	T	Modifier	0.994	0.990	0.976	0.921	0.954	0.972
*SLC6A20*	45760066	rs116638840	C	T	Modifier	0.027	0.076	0.010	-	-	0.003
*SLC6A20*	45762899	rs2191027	C	T	Modifier	0.014	0.020	0.193	0.017	0.299	0.149
*SLC6A20*	45780132	rs2252547	T	C	Modifier	0.155	0.465	0.614	0.450	0.591	0.516

^a^ Reference allele; ^b^ variant allele; * variants without described SNP; (-) no annotation; NAM: Amazonian Native American populations; AFR: African populations; AMR: American populations; EAS: East Asian populations; EUR: European populations; SAS: South Asian populations. All variants described in this table follow the selection criteria based on the impact prediction of modifier, moderate, or high, according to SnpEff.

**Table 3:** Comparison of the allele frequencies between the NAM population and the continental populations (AFR, AMR, EUR, EAS, and SAS).

Gene	SNP ID	NAM vs. AFR *	NAM vs. AMR *	NAM vs. EAS *	NAM vs. EUR *	NAM vs. SAS *
*ABO*	rs55727303	**1.66 × 10^−19^**	**1.08 × 10^−3^**	**1.40 × 10^−17^**	**6.75 × 10^−14^**	**8.82 × 10^−11^**
*ABO*	rs8176748	**1.58 × 10^−19^**	**1.00 × 10^−8^**	**7.06 × 10^−16^**	**1.52 × 10^−20^**	**1.29 × 10^−20^**
*ABO*	rs8176740	**1.58 × 10^−19^**	**1.00 × 10^−8^**	**7.06 × 10^−16^**	**1.52 × 10^−20^**	**1.29 × 10^−20^**
*ABO*	rs8176720	**3.78 × 10^−7^**	**2.10 × 10^−6^**	**4.54 × 10^−7^**	**3.81 × 10^−13^**	**9.84 × 10^−8^**
*ABO*	rs1053878	**6.04 × 10^−7^**	0.149	**0.001**	**0.020**	1.000
*ABO*	rs2073824	**6.48 × 10^−5^**	**5.73 × 10^−4^**	**4.73 × 10^−3^**	**2.04 × 10^−9^**	**5.21 × 10^−5^**
*ABO*	rs559723	**7.94 × 10^−8^**	**6.70 × 10^−4^**	**5.66 × 10^−4^**	**9.77 × 10^−8^**	**1.11 × 10^−11^**
*ABO*	rs616154	**7.78 × 10^−17^**	**5.92 × 10^−11^**	**5.50 × 10^−11^**	**6.58 × 10^−17^**	**3.03 × 10^−22^**
*ABO*	rs2073825	1.000	**3.0 × 10^−3^**	0.382	0.874	0.874
*CCR9*	rs147314165	**4.40 × 10^−4^**	**4.22 × 10^−4^**	**8.63 × 10^−5^**	**8.70 × 10^−5^**	**9.84 × 10^−5^**
*CCR9*	rs7648467	**2.45 × 10^−14^**	0.330	0.113	0.570	0.461
*CXCR6*	rs2234355	**5.37 × 10^−15^**	0.398	**0.035**	0.100	**0.037**
*FYCO1*	rs3733100	**4.13 × 10^−28^**	**3.67 × 10^−5^**	**1.45 × 10^−4^**	**5.81 × 10^−8^**	**5.27 × 10^−3^**
*FYCO1*	rs3796375	**2.35 × 10^−37^**	**4.62 × 10^−5^**	**6.62 × 10^−3^**	**1.20 × 10^−9^**	**3.18 × 10^−12^**
*FYCO1*	rs35678722	**3.39 × 10^−3^**	**4.22 × 10^−4^**	**8.63 × 10^−5^**	**8.70 × 10^−5^**	**9.84 × 10^−5^**
*FYCO1*	rs113517878	**8.44 × 10^−5^**	**4.22 × 10^−4^**	**8.63 × 10^−5^**	**8.70 × 10^−5^**	**9.84 × 10^−5^**
*FYCO1*	rs4683158	0.614	0.008	1.000	**2.49 × 10^−6^**	**0.015**
*FYCO1*	rs13079478	**4.40 × 10^−4^**	0.578	**2.76 × 10^−4^**	0.410	**1.51 × 10^−6^**
*FYCO1*	rs13059238	**0.012**	0.591	**2.76 × 10^−4^**	0.410	**1.51 × 10^−6^**
*FYCO1*	rs33910087	**0.009**	0.578	**0.001**	0.410	**1.51 × 10^−6^**
*FYCO1*	rs1532071	**6.14 × 10^−25^**	**1.36 × 10^−6^**	**1.32 × 10^−5^**	**8.95 × 10^−10^**	**1.82 × 10^−3^**
*FYCO1*	rs3733097	**2.08 × 10^−45^**	**2.84 × 10^−6^**	**6.14 × 10^−4^**	**2.84 × 10^−11^**	**7.34 × 10^−14^**
*FYCO1*	rs751552	**1.41 × 10^−41^**	**1.51 × 10^−4^**	**1.54 × 10^−2^**	**7.09 × 10^−9^**	**2.68 x10^−11^**
*FYCO1*	rs1873002	1.000	1.000	1.000	1.000	1.000
*FYCO1*	rs9875616	0.168	1.000	**6.0 x10^−3^**	**0.045**	0.685
*FYCO1*	rs6800954	**0.012**	0.383	**5.0 × 10^−3^**	0.192	1.000
*FYCO1*	rs41289622	**4.40 × 10^−4^**	0.578	**2.76 × 10^−4^**	0.410	**1.51 × 10^−6^**
*FYCO1*	rs36122610	**0.011**	1.000	**0.012**	0.093	**2.44 × 10^−8^**
*FYCO1*	rs76597151	1.000	0.230	0.302	**5.0 × 10^−3^**	**5.07 × 10^−11^**
*FYCO1*	rs17214952	1.000	0.230	0.302	**5.0 × 10^−3^**	**1.28 × 10^−10^**
*LZTFL1*	rs141398338	**4.40 × 10^−4^**	**4.22 × 10^−4^**	**8.63 × 10^−5^**	**8.70 × 10^−5^**	**9.84 × 10^−5^**
*LZTFL1*	rs138230559	0.712	0.288	0.213	0.213	0.218
*SLC6A20*	rs140440513	**2.59 × 10^−5^**	**4.22 × 10^−4^**	**8.63 × 10^−5^**	**8.70 × 10^−5^**	**9.84 × 10^−5^**
*SLC6A20*	rs17279437	0.371	0.485	0.381	0.05	1.000
*SLC6A20*	rs2252547	**1.08 × 10^−14^**	**6.54 × 10^−12^**	**3.18 × 10^−6^**	**2.12 × 10^−11^**	**1.95 × 10^−8^**
*SLC6A20*	rs2251347	1.000	0.366	**0.016**	0.095	0.380
*SLC6A20*	rs116638840	0.306	0.236	**0.035**	**0.035**	0.068
*SLC6A20*	rs2191027	1.000	**7.90 × 10^−5^**	1.000	**3.58 × 10^−8^**	**1.0 × 10^−3^**

NAM: Amazonian Native American populations; AFR: African populations; AMR: American populations; EAS: East Asian populations; EUR: European populations; SAS: South Asian populations; * *p*-value defined by Fisher’s exact test. Bold characters indicate a significant difference (*p*-value < 0.05).

## Data Availability

The data presented in this study are openly available on Figshare at https://doi.org/10.6084/m9.figshare.18728192.v1; accessed on 25 January 2022.

## Supplementary Materials

The following supporting information can be downloaded at: https://www.mdpi.com/article/10.3390/jpm12040554/s1: Supplementary Table S1: Description of all variants for the genes *ABO*, *CCR9*, *CXCR6*, *FYCO1*, *LZTFL1*, *SLC6A20*, and *XCR1* found in the 64 individuals sampled in the present study.
