# Supplementary material for: The Genomic Profile Associated with Risk of Severe Forms of COVID-19 in Amazonian Native American Populations
Source: J Pers Med. 2022 Apr 1;12(4):554. doi: 10.3390/jpm12040554 (PMC9027999; doi:10.3390/jpm12040554)
Supplement: Supplementary file 1 [file jpm-12-00554-s001.zip › jpm-1591817-supplementary.pdf]

Supplementary Table S1 – Description of all variants for the genes ABO, CCR9, CXCR6, FYCO1, LZTFL1, SLC6A20, and XCR1 found in the 64 individuals sampled in the present study.

Gene	Position	SNP ID	Ref[a]	Var[b]	Impact Predicted by SNPeff	Variant Allele Frequency					
						NAM	AFR	AMR	EAS	EUR	SAS
ABO	133256189	rs55727303	C	T	HIGH	0.281	0.001	0.108	-	0.012	0.026
ABO	133255960	rs8176745	G	A	LOW	0.806	0.244	0.431	0.290	0.221	0.220
ABO	133256050	rs8176742	C	T	LOW	0.814	0.136	0.421	0.289	0.220	0.219
ABO	133256248	rs200932155	G	A	LOW	0.000	0.002	-	-	0.001	-
ABO	133255902	rs8176748	C	T	MODERATE	0.814	0.243	0.431	0.290	0.221	0.220
ABO	133256085	rs8176740	A	T	MODERATE	0.814	0.242	0.431	0.289	0.221	0.220
ABO	133256264	rs1053878	G	A	MODERATE	0.016	0.256	0.068	0.151	0.099	0.029
ABO	133257465	rs8176721	G	A	MODERATE	0.000	0.155	0.017	-	0.005	-
ABO	133257486	rs8176720	T	C	MODERATE	0.814	0.489	0.494	0.483	0.337	0.464
ABO	133257246	rs2073824	A	G	MODIFIER	0.728	0.469	0.496	0.549	0.336	0.465
ABO	133257320	rs2073825	A	T	MODIFIER	0.235	0.241	0.431	0.289	0.222	0.225
ABO	133262062	*	C	A	MODIFIER	0.016	-	-	-	-	-
ABO	133275050	rs616154	C	T	MODIFIER	0.031	0.531	0.408	0.408	0.535	0.631
ABO	133275068	rs559723	A	G	MODIFIER	0.184	0.531	0.408	0.408	0.536	0.631
CCR9	45901310	rs147314165	A	G	LOW	0.083	0.007	-	-	-	-
CCR9	45894830	rs7648467	C	A	MODIFIER	0.014	0.448	0.050	-	0.013	0.008
CCR9	45897524	rs17764980	G	A	MODIFIER	0.000	0.005	0.058	0.004	0.120	0.383
CXCR6	45946488	rs2234355	G	A	MODERATE	0.033	0.491	0.068	-	0.005	0.001
FYCO1	45931236	rs137986696	C	T	LOW	0.180	0.001	0.053	-	-	-
FYCO1	45955269	rs1463680	G	A	LOW	0.986	0.901	0.840	0.954	0.744	0.878
FYCO1	45959401	*	G	A	LOW	0.019	-	-	-	-	-
FYCO1	45959549	rs536305273	CAA	C	LOW	0.000	0.001	0.001	-	-	-
FYCO1	45966595	rs13079869	G	A	LOW	0.070	0.008	0.061	0.004	0.122	0.358
FYCO1	45967024	rs759955437	C	T	LOW	0.009	0.000	0.000	0.000	0.000	0.000
FYCO1	45967999	rs3796376	C	T	LOW	0.000	0.033	0.154	0.293	0.208	0.156
FYCO1	45968128	rs34147726	C	T	LOW	0.023	0.354	0.030	-	-	-
FYCO1	45968515	rs13071283	T	C	LOW	0.070	0.020	0.063	0.004	0.124	0.360
FYCO1	45979726	rs4682801	G	T	LOW	0.963	0.384	0.869	1.000	0.797	0.921
FYCO1	45923752	rs35678722	G	A	MODERATE	0.083	0.012	0.001	-	-	-
FYCO1	45966331	rs13079478	G	T	MODERATE	0.070	0.005	0.059	0.004	0.122	0.360
FYCO1	45966333	rs13059238	T	C	MODERATE	0.070	0.019	0.063	0.004	0.123	0.359

FYCO1	45966722	rs113517878	C	T	MODERATE	0.083	0.003	0.003	-	-	-
FYCO1	45967298	rs3796375	G	A	MODERATE	0.822	0.093	0.565	0.661	0.431	0.372
FYCO1	45967995	rs33910087	G	A	MODERATE	0.070	0.017	0.059	0.005	0.122	0.359
FYCO1	45968372	rs3733100	C	G	MODERATE	0.885	0.210	0.643	0.667	0.556	0.731
FYCO1	45968585	rs4683158	C	T	MODERATE	0.994	0.982	0.914	1.000	0.801	0.922
FYCO1	45979767	rs1306733846	C	T	MODERATE	0.000	0.000	0.000	0.000	0.000	0.000
FYCO1	45923467	rs6800954	C	T	MODIFIER	0.143	0.287	0.193	0.307	0.216	0.148
FYCO1	45936407	rs1873002	T	C	MODIFIER	1.000	1.000	1.000	1.000	1.000	1.000
FYCO1	45938385	rs9875616	G	A	MODIFIER	0.859	0.914	0.850	0.954	0.746	0.879
FYCO1	45959378	rs13069079	G	A	MODIFIER	0.000	0.005	0.058	0.004	0.121	0.371
FYCO1	45959571	rs1532071	G	A	MODIFIER	0.908	0.260	0.614	0.652	0.529	0.732
FYCO1	45959735	rs76597151	G	A	MODIFIER	0.014	0.017	0.062	0.004	0.122	0.371
FYCO1	45969944	rs17214952	A	G	MODIFIER	0.014	0.019	0.063	0.004	0.123	0.360
FYCO1	45973053	rs41289622	T	G	MODIFIER	0.070	0.005	0.059	0.004	0.122	0.361
FYCO1	45975359	rs751552	A	T	MODIFIER	0.814	0.063	0.565	0.661	0.431	0.371
FYCO1	45981341	rs36122610	G	A	MODIFIER	0.054	0.005	0.059	0.004	0.122	0.358
FYCO1	45984767	rs3733097	G	A	MODIFIER	0.853	0.067	0.561	0.655	0.432	0.372
LZTFL1	45828480	rs1129183	C	T	MODERATE	0.000	0.043	0.038	0.039	0.074	0.077
LZTFL1	45827235	*	TCTG	T	MODIFIER	0.016	-	-	-	-	-
LZTFL1	45842023	rs138230559	C	T	MODIFIER	0.009	0.033	-	-	-	-
LZTFL1	45842083	rs141398338	A	C	MODIFIER	0.083	0.005	-	-	0.002	-
SLC6A20	45775836	rs13314717	C	T	LOW	0.083	0.125	0.014	-	0.003	-
SLC6A20	45775926	rs758386	A	G	LOW	0.994	0.972	0.977	0.911	0.954	0.932
SLC6A20	45780081	rs2742399	G	C	LOW	0.016	0.187	0.022	0.004	0.011	0.076
SLC6A20	45759079	rs140440513	C	T	MODERATE	0.083	0.000	0.000	0.000	0.000	0.000
SLC6A20	45759901	rs61731475	T	C	MODERATE	0.000	-	0.006	-	0.014	-
SLC6A20	45772602	rs17279437	G	A	MODERATE	0.017	0.005	0.043	0.006	0.092	0.031
SLC6A20	45775922	rs139429025	T	C	MODERATE	0.000	0.012	-	-	-	-
SLC6A20	45758379	rs2251347	C	T	MODIFIER	0.994	0.990	0.976	0.921	0.954	0.972

SLC6A20	45760066	rs116638840	C	T	MODIFIER	0.027	0.076	0.010	-	-	0.003
SLC6A20	45762899	rs2191027	C	T	MODIFIER	0.014	0.020	0.193	0.017	0.299	0.149
SLC6A20	45780132	rs2252547	T	C	MODIFIER	0.155	0.465	0.614	0.450	0.591	0.516
XCR1	46021837	rs2230322	T	C	LOW	0.109	0.443	0.091	0.047	0.124	0.354

[a] Reference Allele; [b] Variant Allele; * Variants without described SNP; (-) – No annotation; NAM: Amazonian Native American populations; AFR: African population; AMR: American population; EAS: East Asian population; EUR: European population; SAS: South Asian population
